# Perivascular Epithelioid Cell Tumor (PEComa) of the Lung in a 56-Year-Old Female Patient: A Case Report

**DOI:** 10.7759/cureus.29246

**Published:** 2022-09-16

**Authors:** Umberto M Donato, Keith Ferguson

**Affiliations:** 1 Pediatric Oncology, Tampa General Hospital, Tampa, USA; 2 Radiology, Moffitt Cancer Center, Tampa, USA; 3 Pediatric Oncology, USF (University of South Florida) Health, Tampa, USA; 4 Diagnostic Imaging and Interventional Radiology, Moffitt Cancer Center, Tampa, USA

**Keywords:** clear cell tumors, hmb-45 immunohistochemical positivity, clear cell tumor of the lung, coin lesions, immunohistology, mesenchymal tumors, sugar tumor, clear cell, pecoma

## Abstract

Perivascular epithelioid cell tumors, best known as PEComas, are extremely uncommon mesenchymal tumors The etiology of PEComas remains unestablished and its clinical presentation is usually benign. PEComas lack a distinctive symptomatic presentation; thus, the diagnosis of these tumors relies mainly on pathological examinations. These neoplasms have a very distinct immunoreactivity for melanocytic markers critical for their identification. Due to the rarity of these tumors and lack of a distinct disease presentation, we discuss the diagnostic relevance of imaging and pathologic findings in a 56-year-old woman diagnosed with a PEComa in the right middle lobe of the lung.

## Introduction

Perivascular epithelioid cell tumor (PEComa) is an uncommon tumor of mesenchymal origin [1]. This tumor family is characterized by the perivascular distribution of their distinctive epithelioid or spindle cells. PEComas were first identified in 1963 [2] and have since been described in multiple organs, including the kidneys and other genitourinary sites, retroperitoneum, uterus, liver, and lungs. This subset of tumors is now considered to comprise angiomyolipomas (AML), lymphangioleiomyomatosis (LAM), and clear cell sugar tumors (CCST). Most PEComas are benign, although a number of malignant PEComas have been cited in the literature [1]. PEComas are more common in middle-aged individuals and are nearly three times as common in females in comparison to males [3]. Variants of PEComa in the lung include LAM and CCST [3]. The diagnosis of these neoplasms is frequently complicated by the lack of a symptomatic presentation and non-specific imaging features, thus causing physicians to rely mainly on immunohistochemical markers of these tumors [4]. Myogenic and melanocytic markers are commonly reactive and are vital in diagnosing these tumors. Due to the scant evidence of this pathology in the literature and the diagnostic complications associated with it, we present the imaging and histological findings in a 56-year-old woman diagnosed with a PEComa of the lung.

## Case presentation

**Figure 1:**
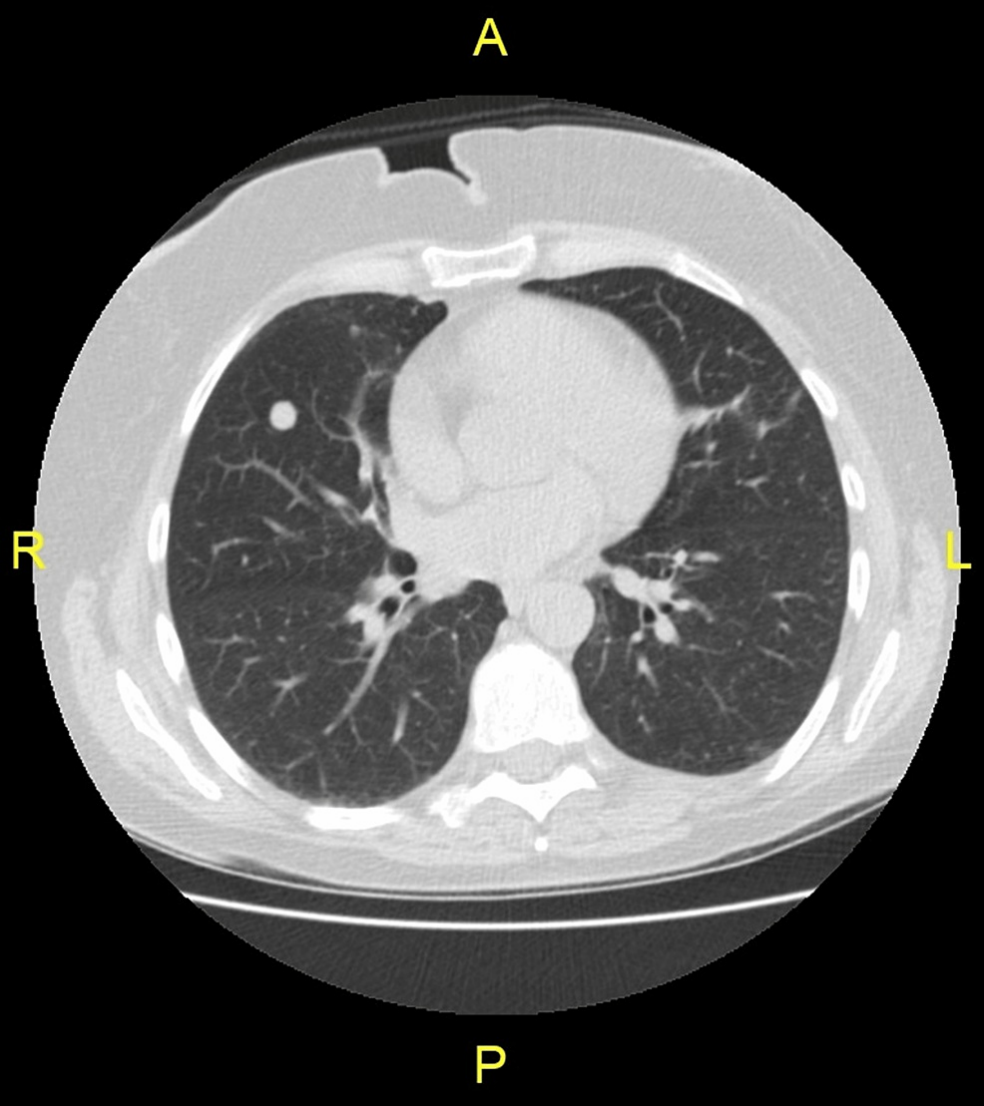
Chest CT findings of a 56-year-old female patient: axial non-contrast CT section through lung base 1 cm diameter right middle lobe nodule

**Figure 2:**
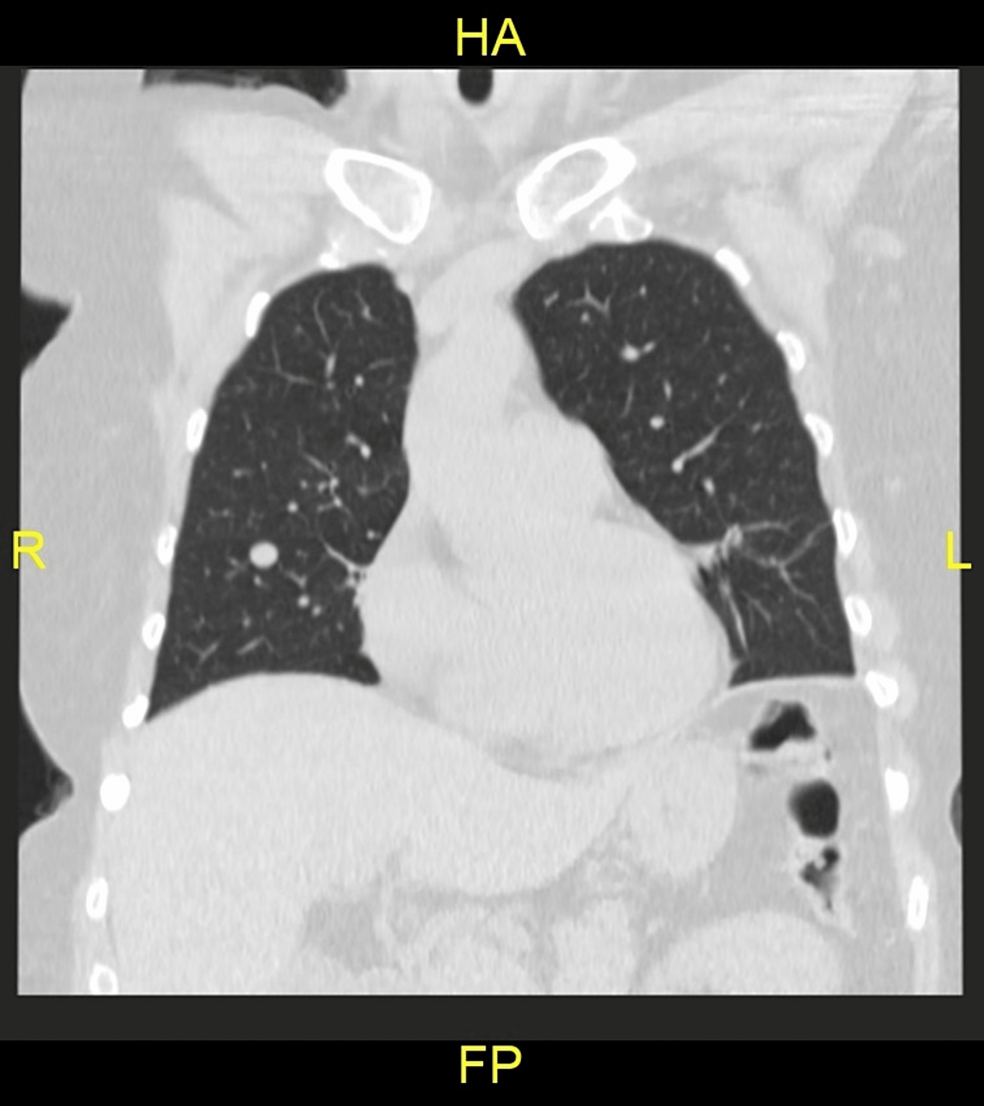
Chest CT findings of a 56-year-old female patient: coronal non-contrast CT section 1 cm diameter right middle lobe nodule

A 56-year-old female smoker (40 pack years), with a history of breast cancer treated with (right-sided) lumpectomy and radiation followed by five years of tamoxifen, presented to her family doctor for evaluation of a right axillary lump. Physical and laboratory results were unremarkable except for a hard 1 cm large fixed nodule in the right axillary region. Due to the smoking history, a low-dose non-contrast screening chest CT was performed and revealed a 1 cm diameter right middle lobe nodule (Figures 1,2) as well as multiple subcentimeter (0.2-0.4 cm) nodules elsewhere in both lungs and right axillary lymphadenopathy.

**Figure 3:**
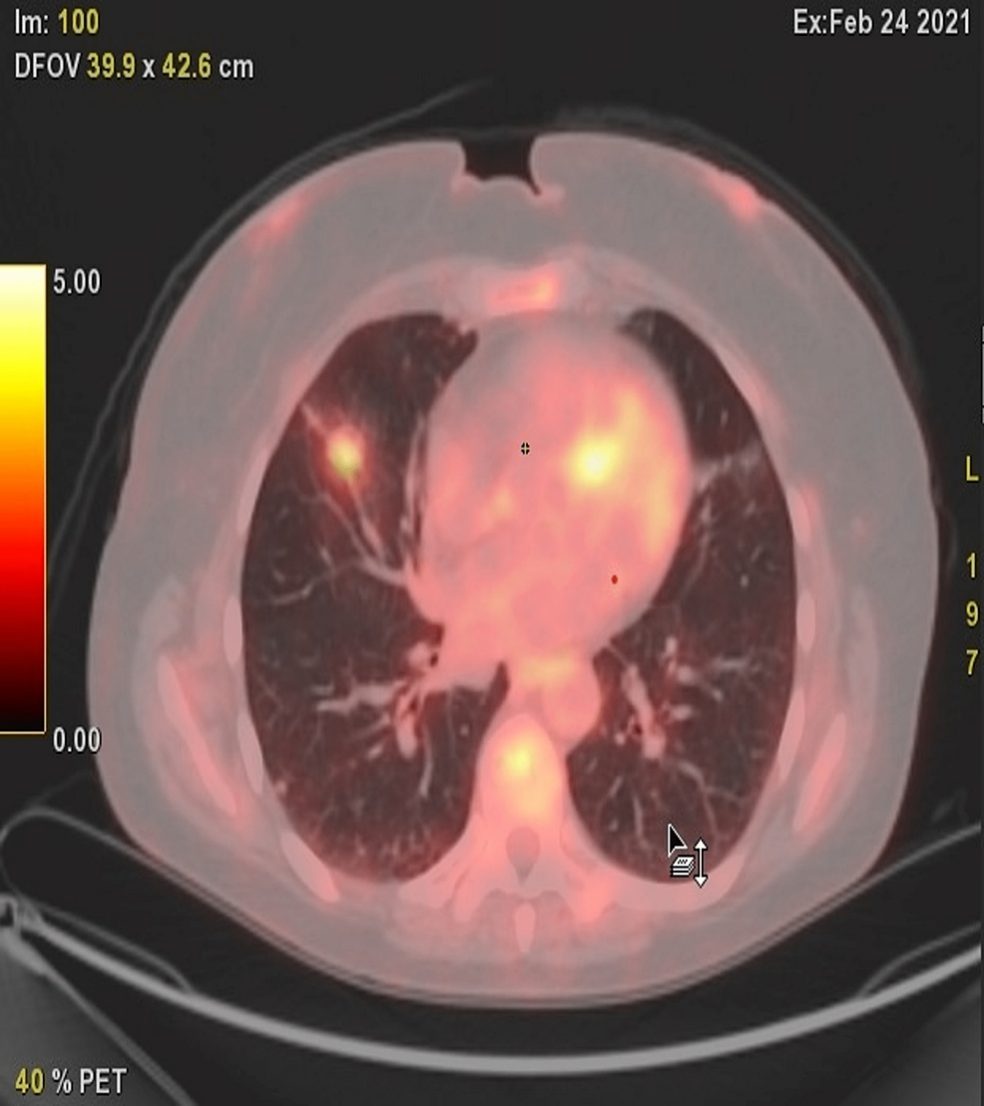
Chest PET-CT findings of a 56-year-old female patient: axial PET-CT section through lung base 1 cm diameter right middle lobe hypermetabolic nodule

She subsequently underwent a CT-guided needle biopsy of the dominant right middle lobe nodule, which was remarkable for a clear cell proliferation suspicious for LAM. The patient underwent a positron emission tomography (PET)/CT one week later, which revealed a 1.5 X 1.1 cm hypermetabolic nodule (Figure 3) along with hypermetabolic right axillary lymph nodes. Considering the patient's history of breast cancer and smoking, the differential diagnosis for these results included metastatic breast cancer and primary lung cancer. Despite the CT-guided needle biopsy proving suspicious for LAM, the patient did not present with symptoms or the pathognomonic parenchymal destruction associated with this disease on imaging, and image-guided biopsy results were, therefore, potentially discordant. In order to provide a definitive diagnosis, the patient underwent thoracoscopic resection of the right middle lobe nodule one month later. Frozen section analysis showed neoplastic proliferation with clear appearing cells suggestive of LAM. Immunohistochemical stains showed lesional cells to be positive for MelanA and focal HMB45. HMB-45 and MelanA, are considered to be the most sensitive melanocytic markers for the diagnosis of PEComa [1]. The final pathology diagnosis was 1.2 cm benign PEComa (CCST) of the lung with no evidence of lymphovascular or visceral pleura invasion and negative margins. In the meantime, the patient underwent further evaluation of the right axillary lymphadenopathy demonstrating metastatic breast carcinoma, for which she received neoadjuvant chemotherapy followed by mastectomy and axillary dissection and then radiation therapy. 

## Discussion

Clear cell tumor of the lung (CCTL), a type of PEComa, usually presents as a singular, asymptomatic nodule. As they are often asymptomatic or present with non-specific symptoms, these tumors present a significant diagnostic dilemma. CCTLs are often detected in routine screenings, such as in the case of our patient, appearing as well-circumscribed “coin” lesions [5]. Due to their rich vascular stroma, they frequently demonstrate prominent enhancement on contrast-enhanced CT [5]. This appearance of a solitary pulmonary nodule is non-specific and can be seen in a wide variety of benign lesions as well as primary and metastatic malignancies. Due to the non-specific clinical and imaging features, pathological evaluation of these tumors is paramount in making the correct diagnosis. CCTLs are frequently misdiagnosed as pulmonary metastases of primary lung or renal clear cell carcinomas [6]. Despite the pathogenesis of these lesions being uncertain, the World Health Organization (WHO) has categorized these neoplasms as unique mesenchymal tumors composed of perivascular epithelioid cells. On routine hematoxylin and eosin (H&E) stains, these tumors demonstrate a “clear cell” appearance due to abundant intracytoplasmic glycogen [7], leading to them also being referred to as “sugar tumors” of the lungs. Mitoses are typically not present [7]. It is vital that these be differentiated from other, malignant clear cell neoplasms such as the clear cell variant of pulmonary adenocarcinoma and metastatic clear cell renal malignancy [8]. Immunohistochemical analysis permits this differentiation as CCTLs demonstrate the presence of melanosomes, perivascular myoid cell antibodies, and HMB-45 reactivity not seen in more aggressive clear cell neoplasms. All these findings were present in our case and are evidenced by literature to be of pericyte origin [9]. Supporting these findings, HMB-45 immunohistochemical positivity has proven to be one of the most reliable and distinctive CCTL diagnostic markers [10-15], although there have been rare documented CCTL lacking HMB-45 positivity [16]. CCTLs are negative for cytokeratin, chromogranin, CD10, and EMA, which help to distinguish them from malignant clear cell tumors (CCTs) [10-15]. Knowing this, our presented case serves to underscore the immunologic variability of this disease and the importance of considering several immunohistochemical markers such as MelanA when clear cell lesions are suspected [1]. 

Despite the majority of the cases being benign, rare malignant instances of primary and metastatic CCTs have been reported [17], and in those cases, a long follow-up period is often suggested. 

The differential diagnoses of CCTs often include carcinoid, granular cell tumor, lung clear cell adenocarcinoma, metastatic malignant melanoma, and renal cell carcinoma [18]. Such cancers can be distinguished by the presence or absence of certain immunohistochemical markers and clinical presentations of the respective diseases. As stated previously, sugar tumors distinguish themselves most frequently via HMB-45 positive, abundant intracellular glycogen, and a negativity for chromogranin and cytokeratin staining. Once the diagnosis is made, surgical resection of the neoplasm with no neoadjuvant therapy or radiation is considered the standard procedure.

## Conclusions

This case report evidences that CCTLs often present with no symptoms and non-specific imaging features. The standard diagnosis of PEComa, specifically CCTLs, is centered on the histopathology and immunohistochemical analysis of the disease. Surgical resection is the standard therapeutic approach to this pathology, and in the vast majority of cases will be curative. 

## References

[1] Kang JB, Seo JW, Park YH, Jang PR (2014). Malignant perivascular epithelioid cell tumor of the uterus with lung metastasis. Korean J Pathol.

[2] Zarbis N, Barth TF, Blumstein NM, Schelzig H (2007). Pecoma of the lung: a benign tumor with extensive 18F-2-deoxy-D-glucose uptake. Interact Cardiovasc Thorac Surg.

[3] Martignoni G, Pea M, Reghellin D, Zamboni G, Bonetti F (2008). PEComas: the past, the present and the future. Virchows Arch.

[4] Okamoto K, Okada Y, Ohno K (2018). A rare case of perivascular epithelioid cell tumor (PEComa) of the greater omentum. World J Surg Oncol.

[5] Seo JB, Im JG, Seo JW, Yeon KM (1996). Clear cell tumor of the lung. AJR Am J Roentgenol.

[6] Gal AA, Koss MN, Hochholzer L, Chejfec G (1991). An immunohistochemical study of benign clear cell ('sugar') tumor of the lung. Arch Pathol Lab Med.

[7] Xu Q, Lu C, Li L, Xu K (2016). Clear cell tumor of the lung: two case reports and a review of the literature. Medicine (Baltimore).

[8] Tsilimigras DI, Bakopoulos A, Ntanasis-Stathopoulos I (2018). Clear cell "sugar tumor" of the lung: diagnostic features of a rare pulmonary tumor. Respir Med Case Rep.

[9] Aragon CJ, Sanches AF, Alacron JP (2005). Benign clear cell tumor of the lung (Article in Spanish). Arc Bronconeumol.

[10] Yan B, Yau EX, Petersson F (2011). Clear cell 'sugar' tumour of the lung with malignant histological features and melanin pigmentation--the first reported case. Histopathology.

[11] Ye T, Chen H, Hu H, Wang J, Shen L (2010). Malignant clear cell sugar tumor of the lung: patient case report. J Clin Oncol.

[12] Kim WJ, Kim SR, Choe YH (2008). Clear cell "sugar" tumor of the lung: a well-enhanced mass with an early washout pattern on dynamic contrast-enhanced computed tomography. J Korean Med Sci.

[13] Harbin WP, Mark GJ, Greene RE (1978). Benign clear-cell tumor ("sugar" tumor) of the lung: a case report and review of the literature. Radiology.

[14] Adachi Y, Kitamura Y, Nakamura H, Taniguchi Y, Miwa K, Horie Y, Hayashi K (2006). Benign clear (sugar) cell tumor of the lung with CD1a expression. Pathol Int.

[15] Sen S, Senturk E, Kuman NK, Pabuscu E, Kacar F (2009). PEComa (clear cell "sugar" tumor) of the lung: a benign tumor that presented with thrombocytosis. Ann Thorac Surg.

[16] Takanami I, Kodaira S, Imamura T (1998). The use of transbronchial lung biopsy to establish a diagnosis of benign clear cell tumor of the lung: report of a case. Surg Today.

[17] Shen L, Lin J, Ren Z, Wang B, Liu Y, Yuan J, Zhan L (2020). Clear cell tumor of the lung could be aggressive: a case report and review of the literature. J Cardiothorac Surg.

[18] Auerbach A, Cassarino DS (2011). Clear cell tumors of soft tissue. Surg Pathol Clin.

